# Bacterial vaginosis: a sexually transmitted disease?

**DOI:** 10.61622/rbgo/2025rbgo78

**Published:** 2025-10-21

**Authors:** Paulo César Giraldo, José Eleutério

**Affiliations:** 1 Universidade Estadual de Campinas Campinas SP Brazil Universidade Estadual de Campinas, Campinas, SP, Brazil.; 2 Universidade Federal do Ceará Fortaleza CE Brazil Universidade Federal do Ceará, Fortaleza, CE, Brazil.

There is a tendency among health professionals to consider bacterial vaginosis (BV) as a sexually transmitted disease, which implies the treatment of sexual partners.^(1)^ This premise, however, lacks unequivocal confirmation since, in addition to subjecting the sexual partner(s) to questionable treatment (side effects of the medication, increased bacterial resistance), it may also suggest that the disease may have been acquired through sexual relations with a third person. Information lacking clear scientific evidence may lead to marital problems and a depreciation of self-esteem.^(2)^

BV is a syndrome characterized by a greyish-white discharge, often accompanied by an unpleasant smell, and without clinical inflammation.^(2)^ From a microbiological perspective, there is a significant increase in anaerobic bacteria and a notable absence of lactobacilli.^(3)^ Despite this awareness, it has not yet been possible to determine the cause of this alteration in the vaginal ecosystem. Unlike syphilis, trichomoniasis and gonorrhea, there is no single agent that can be held responsible for the clinical presentation, suggesting that the signs and symptoms are a consequence of vaginal dysbiosis with alterations in vaginal acidity and not the action of a single triggering agent.

The bacteria involved in BV produce mucinases, proteases, and sialidases,^(4)^ with an increase in trimethylamine (responsible for the unpleasant vaginal odor), which, associated with the lack of lactic acid-producing lactobacilli, determines the clinical presentation of BV. Despite knowing that different bacterial communities produce these substances, the scientific texts that attempt to suggest that BV is a sexually transmitted infection disregard the immune response of the vaginal epithelium (substances resulting from the metabolism of epithelial cells, defense cells and transudation of the vaginal mucosa) in this process.^(5)^

It does not seem logical to recommend treatment for sexual partners of women with BV to solve an endogenously generated problem. The same reasoning should be considered for same-sex partners.

Rationale for considering that BV is not a sexually transmitted infection:

- The causal agent of BV has not been isolated or identified.
- It is a vaginal dysbiosis, similar to what also occurs in intestinal dysbiosis.
- The bacteria present in BV are the same as those that are part of the vaginal ecosystem, with merely an imbalance between aerobic and anaerobic bacteria.
- In most studies in which the sexual partner was treated, there was no significant improvement in the disease or the recurrence over a long period (6 months or more).
